# Variation in disease phenotype is marked in equine trypanosomiasis

**DOI:** 10.1186/s13071-020-04020-6

**Published:** 2020-03-21

**Authors:** Alexandra G. Raftery, Saloum Jallow, Robert M. Coultous, Jean Rodgers, David G. M. Sutton

**Affiliations:** 1 The Weipers Centre Equine Hospital, Large Animal Clinical Sciences and Public Health, School of Veterinary Medicine, College of Medical, Veterinary and Life Sciences, University of Glasgow, Bearsden Road, Glasgow, UK; 2 Gambia Horse and Donkey Trust, Sambel Kunda, Central River District The Gambia; 3 Institute of Biodiversity, Animal Health & Comparative Medicine, College of Medical, Veterinary & Life Sciences, University of Glasgow, Glasgow, UK

**Keywords:** Equine, Horse, Donkey, Trypanosomiasis, Trypanosomes, Disease phenotype, Co-infections

## Abstract

**Background:**

Equine trypanosomiasis is a severe and prevalent disease that has the greatest impact globally upon working equids due to its distribution across lower income countries. Morbidity and mortality rates are high; disease management strategies in endemic regions are ineffective and cost prohibitive. Individual variation in disease phenotype in other species suggests host factors could reveal novel treatment and control targets but has not been investigated in equids.

**Methods:**

A prospective clinical evaluation of equines presenting for a free veterinary examination was performed in hyperendemic villages in The Gambia. Age, body condition score and body weight were estimated by validated methods, and haematocrit and total protein concentration measured. Animals fulfilling 2 out of 5 clinical inclusion criteria (anaemia, poor body condition, pyrexia, history of abortion, oedema) for a diagnosis of trypanosomiasis received trypanocidal treatment with follow-up at 1 and 2 weeks. Blood samples underwent PCR analysis with specific *Trypanosoma* spp. primers and results were compared to the subject’s clinical and clinicopathological features. A mixed effects generalised linear model was generated to evaluate the association of infection status with degree of pyrexia and anaemia.

**Results:**

Morbidity was high within examined (*n* = 641) and selected (*n* = 247) study populations. PCR status was not associated with a defined disease phenotype; there was intra- and inter-species variability. Donkeys were more frequently *Trypanosoma* spp.-positive (*P* < 0.001) and febrile (*P* < 0.001) than horses, but infected horses were more anaemic (*P* < 0.001), and in poorer body condition (*P* < 0.001) than donkeys. Sex was correlated to disease phenotype: males were more anaemic (*P* = 0.03) and febrile (*P* < 0.001). Haemoparasite co-infections were more common than a single infection.

**Conclusions:**

There was evidence of diversity in trypanosomiasis clinical signs plus variable disease phenotypes within equid subpopulations that warrant further investigation. The complex co-infection profile of field cases requires greater consideration to optimise disease management.
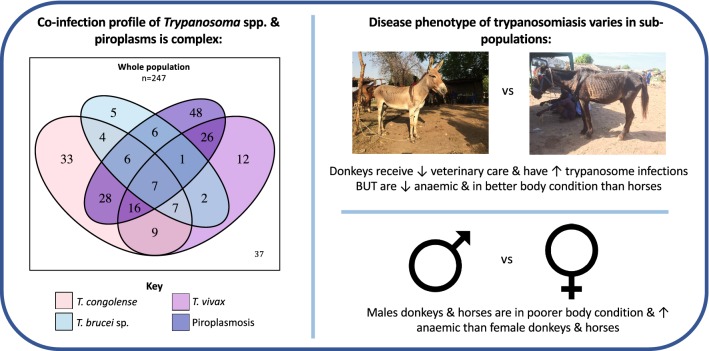

## Background

Globally, working equids have a continued and growing socioeconomic role in supporting the livelihoods of between 300–600 million people, often within the most vulnerable communities [1]. Working equids contribute significantly to household income [2–4], are utilised for transport and traction [5], and create opportunities for women and children [3, 6]. The world equid population (mules, donkeys and horses) is estimated to be just over 127 million (FAO, 2014 [7]) and approximately 85% are working equids in low income countries like The Gambia [8]. The positive impact of working equids upon poverty reduction, sex equality and environmental stability [9, 10] provides impetus to tackle the myriad of issues hindering their welfare and productivity.

Infectious diseases are a major inhibitor of welfare and productivity in this population [1, 9–11] and equine trypanosomiasis is a priority disease with almost global distribution for which strategies are required to improve diagnosis, management and treatment [10]. Equine trypanosomiasis is hyperendemic in The Gambia [12–15] and mortality is high [16]. The Gambia is typical of countries in the tsetse belt with respect to the mix of equid species, husbandry practice and concurrent disease and management problems [16–21].

Available disease control strategies are limited and ineffective [22]. Individual variation in disease phenotype in other species suggests that host factors could reveal novel treatment and control targets [23–25]. Asymptomatic or subclinical individuals constitute a unique resource to investigate the mechanisms underlying control of infection in hosts and offer an additional disease management strategy through selective breeding programmes [23–25]. For population level disease control programmes, subclinical or asymptomatic animals are also an important consideration as they act as a parasitic reservoir.

Previous studies support the presence of subclinical equid disease and greater resilience of donkeys compared to horses. In The Gambia *Trypanosoma* spp. prevalence (by PCR) was similar in a random sample of equids with no concerns noted by owner to those presenting for veterinary examination, however clinical examination findings were not reported [12]. Asymptomatic wandering donkeys infected with *Trypanosoma vivax* have also been identified in Brazil [26] and in sampled equid populations prevalence of disease in donkeys has been reported to be lower than in horses [12, 14, 15, 27]. In horses, higher infection rates have been reported in female horses and horses > 1 year-old [14]. These variations in equine trypanosomiasis phenotype have not been further explored, specifically evaluating in more detail, the variation in the presence and severity of clinical signs in addition to anaemia, such as body condition score and pyrexia (using species-specific reference), factors which significantly impact welfare and ability to work effectively. This information is important to generate evidence for whether an asymptomatic or subclinical status is present in subpopulations of equines and if so, what clinical signs are moderated.

Therefore, the aim of this study was to further characterise the trypanosomiasis disease syndrome in equids through collation of detailed clinical, clinicopathological and diagnostic information. It was hypothesised that disease expression would vary between animals and that subpopulations would have differing severity of disease.

## Methods

### Recruitment of study participants

Subjects were recruited at two time points (November 2012 and 2013) from 10 villages in the Central River District in The Gambia. At each village, recruitment was *via* a one-day mobile veterinary clinic. All owners of equids were invited through notification of the visit through the village chief to attend either for a free health check or due to pre-existing health concerns and were examined by an experienced equine vet (AGR, DGMS). A detailed history following a standardised format was obtained *via* Gambia Horse and Donkey Trust staff acting as translators. The history and clinical findings were recorded on a dedicated form (Additional file 1: Figure S1). Age was estimated from dentition [21], body condition (0–5/5) was scored [21, 28] and body weight (kg) was estimated using a validated nomogram [28–30]. A 6 ml jugular blood sample was taken *via* direct venepuncture for measurement of haematocrit (HCT %) and total plasma protein (TP g/l). Excess blood was stored in EDTA, initially at 4 °C. For animals subsequently included in the treatment trial, 0.2 ml of whole blood was placed upon a Whatman® FTA Classic card (GE Healthcare, Chicago, USA), as a contingency for lost or damaged whole blood samples, and the remainder of the EDTA blood tube was stored at − 20 °C for later PCR analysis. Excess blood was discarded after measurement of HCT and TP if the animal was not included in the study.

Animals were considered eligible for inclusion in the study if two of five clinical criteria, indicative of trypanosomiasis were fulfilled. These clinical criteria were: body condition score ≤ 1.5/5; HCT ≤ 24%; pyrexia (horse > 38.5 °C; donkey > 37.8 °C); limb or ventral oedema; and a history of abortion. Animals were excluded from the study group if they had a concurrent debilitating disease (such as a fracture or severe soft tissue injury), primary presenting sign of neurological disease or had received trypanocidal treatment in the previous month.

### Enrolment and treatment

For included animals a microchip with a unique identifying number was placed in the mid-third of the nuchal ligament at the time of inclusion. The animal was randomly assigned to a treatment group (melarsomine dihydrochloride, diminazene or isometamidium) using simple randomisation that was concealed from those enrolling animals onto the study. Drug doses and routes of administration are detailed in Additional file 2: Table S1.

Animals were re-evaluated at 1 and 2 weeks post-treatment, with further history and blood sample collection. If repeat evaluation was consistent with a clinical diagnosis of severe trypanosomiasis (two out of three remaining of anaemia ≤ 24%, pyrexia (horse > 38.5 °C; donkey > 37.8 °C) or dull demeanour) i.e. a poor clinical response, the treatment was repeated with a different trypanocide (isometamidium or if used previously, diminazene) at the time of the second re-examination. This choice was based upon the current preferences of livestock agents for trypanocidal use in The Gambia.

### Sample processing

Centrifugation (SpinCrit Centrifuge®; E. Brown, Indianapolis, United States) of micro-haematocrit capillary tubes was used to measure HCT. TP was measured on plasma using a refractometer that had been pre-calibrated with deionised water.

DNA extraction and PCR analysis for *Trypanosoma* sp. was performed as previously outlined ([31], Additional file 3: Text S1) using highly sensitive and specific primers targeting multicopy satellite DNA target sequences [32]. The pre-treatment blood samples, taken during the initial examination on week 1, were also screened for piroplasmosis using a modified *Babesia*/*Theileria 18S* catch-all primer set in a nested PCR assay [33–35]. A subsection of the week 1 samples (*n* = 60) was also screened for *Anaplasma* spp. as another potential cause of anaemia using a catch all primer set and nested PCR as previously described [36].

### Interpretation of clinical and clinicopathological data

Species specific reference ranges were used during data analysis. The values applied for temperature, heart rate, respiratory rate [37, 38] and haematocrit [39, 40] are detailed in Additional file 4: Table S2.

### Statistical analysis

Rstudio® and R version 3.3.2 was used for statistical analysis. Normality testing was performed with the Shapiro-Wilk test. Median and inter-quartile range were reported for numerical non-parametric data; mean and standard deviation for parametric data. The one sample t-test for proportion was used to compare proportions from the same population. The Wilcoxon signed rank-test was used for comparison of paired non-parametric data and the Wilcoxon rank-sum test for independent non-parametric data. Chi-square test was used to compare two proportions of categorical data. For comparison of paired proportions, the McNemarʼs test was used. A mixed effects generalised linear model was generated with degree of pyrexia or anaemia as the outcome variable and village as a random effect using backwards elimination to maximise the fit of the model. The impact of variables on model fit was assessed with ANOVA. The level of significance was set as *P* ≤ 0.05. Where data were missing, parameters were reported as a proportion and/or percentage of the number of animals for which the information was available.

## Results

**Fig. 1:**
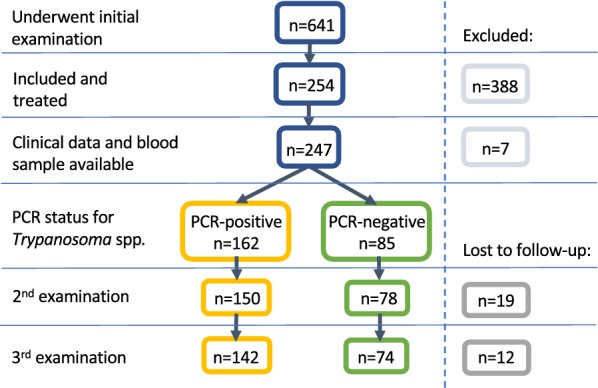
Flow chart illustrating recruitment of equines from the sampled (*n* = 641) to the study population (*n* = 247)

Data from the sampled population (*n* = 641) were used to describe the Gambian equid population. Animals from the study population that had both clinical disease criteria and PCR data (*n* = 247) were used to evaluate clinical descriptors and predictors of disease (*Trypanosoma* spp.) (Fig. 1).

### Description of the sampled population

**Fig. 2:**
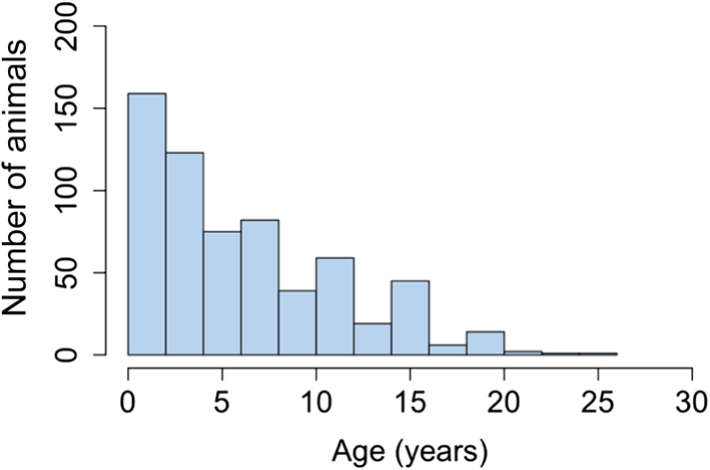
Histogram of the estimated age (years) of the sampled population (*n* = 641) illustrating positive skew of data distribution

In total 641 equids (279 horses, 362 donkeys) were examined (Additional file 5: Table S3) with a marginally greater proportion of female animals (54%; *t*_(640)_ = 2.032, *P* = 0.04). Median estimated age was young (5 years-old, range 2–10 years) (Fig. 2). Access to previous veterinary treatment (368/641, 57%) and specifically trypanocidal treatment within the previous year (223/641, 35%) was limited. Absence of treatment within the last year was more common in donkeys than horses (*χ*^2^ = 49.41, *df* = 1, *P* < 0.001). A history of at least one previous abortion was common (60/342; 18% females) in this population. Clinical abnormalities in the general sampled population were extremely common, resulting in a high proportion of animals being selected for study inclusion (247/641, 39%) of which a significant proportion exhibited 3 or more of the criteria (38%, 93/247).

### Morbidity was high in the study population

The selected study population consisted of 247 animals (106 horses, 141 donkeys) (Fig. 1). Demographic and clinical data describing the study population are summarised in Table 1. Less than half of the study animals had received any previous veterinary treatment (111/247, 45%) or undergone specific treatment for trypanosomiasis (92/247, 37%) within the last year. Seventy percent of owners (158/226) of animals selected for treatment reported a concern regarding health with the remainder not expressing a concern for health or work output. The history of abortion was higher in the females of the selected study population (24%, 35/144) but not significantly greater (*χ*^2^ = 2.84, *df* = 1, *P* = 0.091) than in the general sampled population (18%, 60/340), despite being one of the inclusion criteria. In the selected study population female animals (58%, 144/247) were more common than males (42%, 103/247, *t*_(246)_ = 2.646, *P* = 0.009). The median age within the group was 6 years-old (range 2.5–12 years) with a positive skew to the data distribution, such that the study population was marginally but significantly older than the general population sample (*U* = 68410, *P* = 0.018). The selected study population showed poor body condition scores (BCS) (median BCS 1.5/5) and subdued demeanour (quiet, alert and responsive (QAR): 136/224, 61%; dull: 48/224, 21%) was a frequent finding. Elevations in clinical parameters were common; 51% (126/247) of selected animals were pyrexic, 74% (182/247) tachycardic and 55% (137/247) tachypnoeic. Most animals in the study population were anaemic (232/247, 94%) according to species specific reference ranges (median HCT 22%, IQR 18–24). Median total plasma protein (78 g/l) was at the upper end of the reference range (70–88 g/l).

**Table 1:** Demographics, history and clinical examination findings of the selected study population (*n* = 247)

	Study population (*N* = 247)	Donkeys (*N* = 141)	Horses (*N* = 106)
Demographic data			
Age (years), median (IQ range)	6 (2.5–12.0)	6 (2.5–10.0)	8 (2.5–14.0)
Female, *n*/*N* (%)	144/247 (58)	81/141 (57)	63/106 (59)
Male, *n*/*N* (%)	103/247 (42)	60/141 (43)	43/106 (41)
Estimated weight (kg), median (IQ range)	150 (120–225)	121 (110–145)	240 (200–260)
History			
No veterinary treatment > 1 year, *n*/*N* (%)	130/247 (53)	94/141 (67)	36/106 (34)
No trypanocidal treatment for > 1 year, *n*/*N* (%)	149/247 (60)	105/141 (74)	44/106 (42)
Presented only for health check, *n*/*N* (%)	68/226 (30)	49/133 (37)	19/93 (20)
Abortion, *n*/*N* (%)	35/144 (24)	17/81 (21)	18/63 (29)
Diarrhoea, *n*/*N* (%)	10/247 (4)	1/141 (1)	9/106 (8)
Recent/ recurrent colic, *n*/*N* (%)	4/247 (2)	1/141 (1)	3/106 (3)
Body condition score (0–5/5), median (IQ range)	1.5 (1.5–2.0)	1.5 (1.5–2.0)	1.5 (1.0–1.5)
0.5, *n*/*N* (%)	18/243 (7)	2/139 (1)	16/104 (15)
1, *n*/*N* (%)	42/243 (17)	19/139 (14)	23/104 (22)
1.5, *n*/*N* (%)	109/243 (45)	61/139 (44)	48/104 (46)
≥ 2.0, *n*/*N* (%)	74/243 (30)	57/139 (41)	17/104 (17)
Demeanour			
BAR, *n*/*N* (%)	40/224 (18)	28/124 (22)	12/100 (13)
QAR, *n*/*N* (%)	136/224 (61)	74/124 (58)	62/100 (65)
Dull, *n*/*N* (%)	48/224 (21)	22/124 (17)	26/100 (27)
Parameter			
Temperature (°C), median (IQ range)	38.1 (37.7–38.6)	38.2 (37.8–38.7)	37.8 (37.4–38.5)
Pulse (bpm), median (IQ range)	56 (48–64)	60 (52–68)	48 (42–60)
Respiration (bpm), median (IQ range)	32 (24–46)	38 (28–48)	30 (24–40)
Haematocrit (%), median (IQ range)	22 (18–24)	21 (16–24)	22 (19–25)
Total plasma protein (g/l), median (IQ range)	78 (70–88)	80 (72–88)	78 (70–88)

*Notes*: Data are presented as median values (interquartile range) or proportions (percentages)

*Abbreviations*: BAR, bright, alert and responsive; QAR, quiet, alert and responsive; IQ, interquartile, n, number of animals with variable present; N, number of animals with variable measured; bpm, breaths or beats per minute

### Complex infection patterns were common

**Fig. 3:**
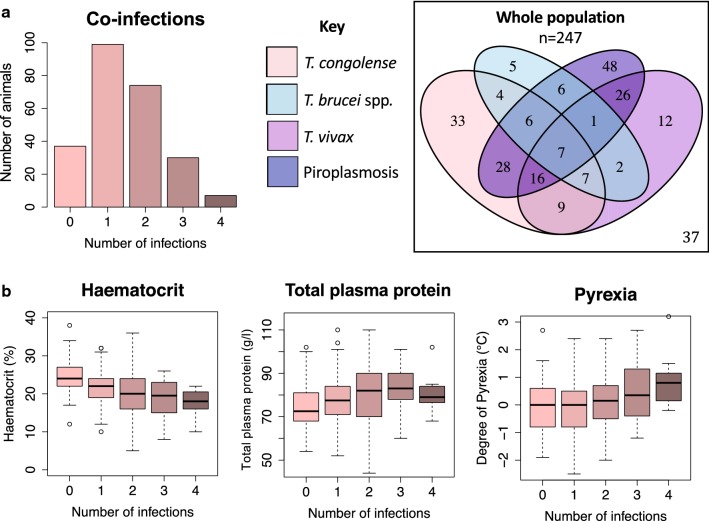
Co-infection status (trypanosomes and piroplasms) of the study population (*n* = 247) and correlation with clinical parameters. **a** Bar plot illustrating the number of animals (*n* = 247) with the different number of co-infections of haemoparasites (*T. brucei*, *T. congolense*, *T. vivax*, piroplasmosis). Venn diagram illustrating the complex and varied infection status of the study population (*n* = 247). Co-infections with multiple haemoparasites were common. A small proportion of animals (15%, 37/247) were negative in PCR for both *Trypanosoma* spp. and piroplasmosis (*Theileria equi*/*Babesia caballi*). **b** Boxplots demonstrating haematocrit (%), pyrexia (°C) and total plasma protein (g/l) of animals with increasing number of co-infections. For each parameter the median (bold line); upper and lower IQR (upper and lower box boundary), upper whisker = min(max(x), Q3 + 1.5 × IQR), lower whisker = max(min(x), Q1 − 1.5 × IQR) where IQR = − Q3 − Q1, the box length. o indicates outliers outside the whiskers

Sixty-six percent (162/247) of the selected animals were positive for at least one *Trypanosoma* sp. by PCR. More than half (55%, 137/247) of the animals were also positive for piroplasmosis (sequencing was consistent with *Theileria equi*, data not shown). Within this population *T. congolense* was the most common trypanosome species detected (110/247, 45%) followed by *T. vivax* (80/247, 32%) and *T. brucei* (38/247, 15%). Co-infections were more common (112/247, 45%) than single trypanosome infections (98/247, 40%) (Fig. 3). None of the tested animals (0/60, 0%) were positive for anaplasmosis.

### *Trypanosoma* spp. PCR status did not define a clear disease phenotype

**Fig. 4:**
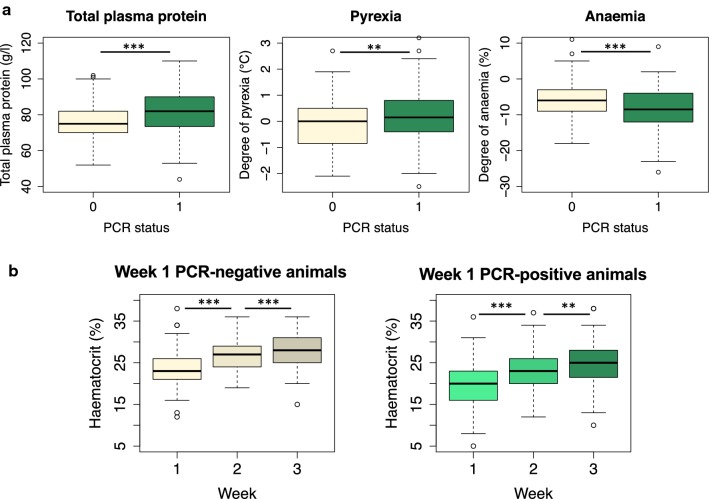
*Trypanosoma* spp. PCR status did not define a clear disease phenotype. **a** Boxplots illustrating significant differences in clinical and clinicopathological parameters between PCR *Trypanosoma* spp.-positive (*n* = 162) and PCR *Trypanosoma* spp. negative (*n* = 85) animals. Degree of pyrexia (*P* = 0.007), degree of anaemia (*P* < 0.001) and total plasma protein (*P* < 0.001) did differ significantly but these parameters were not discriminatory for infection status. Degree of pyrexia (°C) was calculated as the difference between the animalʼs rectal temperature and the upper reference value (horse 38.5 °C; donkey 37.8 °C). Degree of anaemia (%) was calculated as the difference between the animal’s HCT and the lower reference value (horse 31%; donkey 27%). **b** Boxplots illustrating the increases in HCT noted in both the *Trypanosoma* spp. PCR-negative population (*n* = 85) and *Trypanosoma* spp. PCR-positive population (*n* = 162) from the initial evaluation (week 1) and the two follow-up evaluations (week 2, week 3) after treatment with a trypanocidal drug. Piroplasmosis status did not significantly impact on this response to trypanocidal treatment. ***P < 0.001, **P < 0.01, *P < 0.05

Baseline parameters of demeanour, body condition score, heart rate and respiratory rate were comparable (*P* > 0.05) between *Trypanosoma* spp. PCR-positive and negative animals (Additional file 6: Table S4). Degree of pyrexia (*U* = 8226, *P* = 0.007), anaemia (*U* = 4829, *P* < 0.001) and total plasma protein concentration (*U* = 8586, *P* < 0.001) differed significantly between *Trypanosoma* spp. PCR-positive and negative animals; however, these parameters were not discriminatory for infection status based on PCR findings (Fig. 4a).

### Evidence for *Trypanosoma* spp. infection in the PCR negative population

**Fig. 5:**
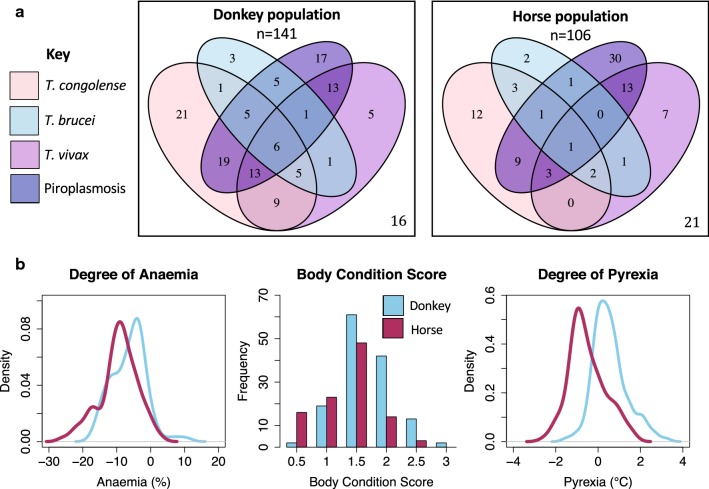
Variation in *Trypanosoma* spp. disease phenotype between donkeys and horses. **a** Venn diagrams illustrating complex co-infection status of the study population (*n* = 247), separated into donkeys and horses. Donkeys were more frequently co-infected with *Trypanosoma* spp. and/or piroplasmosis than horses (*P* < 0.001). **b** Differing clinical parameters by equine species. The density plot of species adapted degree of anaemia highlights the greater degree of anaemia noted in horses (maroon) than donkeys (blue) (*P* < 0.001). The reference ranges used were: donkey 27–42%; horse 31–43%. The bar plot illustrates the difference in data distribution for body condition. The density plot of relative degree of pyrexia (°C) highlights the higher rectal temperature of donkeys (blue) than horses (maroon) in relation to the accepted reference range for each species (*P* < 0.001). The reference ranges used were: donkey 36.2–37.8 °C; horse 37.5–38.5 °C

Within the population selected based on clinical presentation for trypanosomiasis (*n* = 247), assessment of the impact of trypanocidal treatment upon anaemia further supported the initial diagnosis that this cohort (including the PCR-negative animals) was likely to be infected and affected by trypanosomes since HCT improved post-trypanocidal treatment irrespective of PCR status of the animal (Fig. 4b). This assumption was made after consideration of other potential causes of this response. This includes other potentially endemic infectious causes of anaemia (anaplasmosis, piroplasmosis, equine infectious anaemia, African horse sickness) or an inflammatory response to the trypanocidal drug resulting in bone marrow stimulation. Available data and clinical examinations support that equine infectious anaemia and African horse sickness are not circulating in The Gambia [41]. A random subsection of samples (*n* = 60) were negative on a catch all primer for anaplasmosis. Further diagnostics identified that piroplasmosis is endemic (55%; Fig. 5) in this population. In endemic populations the pathogenic impact is reduced, as for most animals, immunity is generated [42]. This was supported by a mixed effect GLM model with HCT as the outcome variable where piroplasmosis status was not correlated with HCT (Additional file 7: Table S5). The positive response to trypanocides was still present when piroplasmosis-positive samples were removed from the analysis and when diminazene treated animals were removed (to which piroplasms have some susceptibility [43, 44]). It would be unlikely for there to be an inflammatory response capable of causing consistent bone marrow stimulation to all three trypanocides.

It was concluded that the clinical disease criteria (i.e. clinical signs of trypanosomiasis) used to select these animals, the documented high prevalence of *Trypanosoma* spp. in this region [12, 14, 15], known low parasitaemia of chronic trypanosome infection and positive response to trypanocidal medication significantly increase the likelihood that the negative PCR result is due to a parasitaemia below a threshold of detection (this is an assumption made for the subsequent analysis). Therefore, for the purposes of this analysis this whole population (*n* = 247) was used as a trypanosome-positive population (with PCR status providing a threshold of parasitaemia) to investigate clinical predictors and descriptors of trypanosome infection.

### Multiple factors were associated with pyrexia

Pyrexia results in dullness and lethargy and is commonly correlated to increasing trypanosome parasitaemia, and is therefore, important clinically with impacts both on welfare and productivity. A mixed effects generalised linear model (GLM) was used to identify factors associated with the clinical outcome of pyrexia (Additional file 8: Table S6) in the selected study population (*n* = 247) whilst controlling for the mixed effect of location (village) on the variance of pyrexia. There is no assumption of causality within the model (or the model for anaemia) but correlations are useful to guide clinical reasoning in pathophysiology.

Increasing heart rate was significantly correlated to temperature: an increase of 10 bpm was associated with an increase in temperature by 0.3 ± 0.04 °C (coefficient ± standard error). Male sex was associated with a higher (0.4 ± 0.1 °C) rectal temperature than female sex. Horses were less (− 0.9 ± 0.1 °C; *P* < 0.001) pyrexic than donkeys. The only haemoparasite infection that was correlated to pyrexia was *T. brucei*; positive PCR status (*P* = 0.046) was associated with an 0.3 ± 0.1 °C increase in temperature. Respiratory rate was related to temperature (*P* < 0.001), an increase of 10 bpm was associated with an increase in temperature by 0.2 ± 0.004 °C. Increasing estimated age had a small negative coefficient of 0.2  ± 0.08 °C per 10 years (*P* = 0.003); this was expected as reference ranges were not adjusted for the higher normal reference range for temperature in young animals. The geographical location (village number) made a small contribution (variance 0.03; standard deviation 0.17) to the total variation in temperature (residual variance 0.4; standard deviation 0.7). Piroplasmosis PCR status and number of co-infections did not have a significant correlation with pyrexia.

### Multiple factors were associated with anaemia

Anaemia results in lethargy and may be life threatening or performance limiting in equids depending on severity. Control of anaemia is a trypanotolerant trait. It is therefore important clinically with impacts both on welfare and productivity. A mixed effects generalised linear model identified multiple factors significantly associated with degree of anaemia (Additional file 7: Table S5) in the selected study population (*n* = 247). Heart rate was associated with anaemia (*P* < 0.001) but the coefficient was small, an increase of 10 bpm was associated with a decrease in haematocrit by 0.7 ± 0.03%. Horses were more anaemic (− 3 ± 0.7%) than donkeys (*P* < 0.001). The three *Trypanosoma* spp. were correlated to anaemia; *T. brucei* infection had the greatest coefficient with an associated decrease in HCT of 2.3 ± 0.9% (*P* = 0.003), followed by *T. congolense* (− 1.9 ± 0.6%; *P* = 0.003) then *T. vivax* (− 1.7 ± 0.65%; *P* = 0.01). Animals with a dull demeanour on clinical examination were more anaemic (− 2.9 ± 1.0%; *P* = 0.005) than those with brighter (BAR or QAR) demeanour. The geographical location (village number) made a small contribution (variance 3.7; standard deviation 1.9) to the total variation in degree of anaemia (residual variance 17.6; standard deviation 4.2). Piroplasmosis PCR status and number of co-infections did not remain in the model and therefore did not have a significant correlation with degree of anaemia in this population.

### Trypanosome infected horses were more severely affected than donkeys

#### Veterinary care was sought more frequently for horses

Horses were more likely to have received previous veterinary treatment (*χ*^2^ = 25.96, *df* = 1, *P* < 0.001) and specifically trypanocidal treatment (*χ*^2^ = 27.463, *df* = 1, *P* < 0.001) compared to the donkey population in the region. Owners reported clinical abnormalities more commonly in horses than in donkeys at the time of examination (*χ*^2^ = 7.008, *df* = 1, *P* = 0.008) (Table 1).

#### Donkeys were more frequently Trypanosoma spp.-positive

There was a greater proportion of donkeys than horses that were PCR-positive for at least one *Trypanosoma* spp. (*χ*^2^ = 15.4, *df* = 1, *P* < 0.001). A greater proportion of donkeys were PCR-positive for *T. congolense* compared to horses (*χ*^2^ = 17.6, *df* = 1, *P* < 0.001) (Fig. 5) but for all other trypanosome species there was only a trend towards significance. Co-infections of *Trypanosoma* spp. and piroplasmosis were more common (*χ*^2^ = 11.0, *df* = 1, *P* < 0.001) in donkeys (79/125, 63%) than in horses (34/85, 40%) (Fig. 5). There was no difference in the proportion of horses or donkeys infected with piroplasmosis in the group selected on the basis of clinical signs.

#### Body condition was lower in horses

Horses meeting the inclusion criteria had a significantly lower body condition score (*U* = 4586, *P* < 0.001) than donkeys. Furthermore, donkeys were also less likely to have a very poor body condition score (≤ 1/5) (*χ*^2^ = 15.8, *df* = 1, *P* < 0.001) and were more likely to have an acceptable body condition score (≥ 2/5) than horses (*χ*^2^ = 17.2, *df* = 1, *P* < 0.001) (Table 1, Fig. 5b).

#### Horses were more anaemic

There was no significant difference in absolute values of haematocrit between donkey and horse (*U* = 8368, *P* = 0.11). Median values for both species [donkey 21% (16–24%); horse 22% (19–25%)] were below reported reference ranges. However, when the degree of anaemia was evaluated using the absolute percentage points below the lower reference range, horses were significantly more anaemic than donkeys (*U* = 5224, *P* < 0.001) (Fig. 5b).

#### Donkeys were more febrile

The median rectal temperature of donkeys was higher [38.2 °C (37.8–38.7 °C)] than horses [37.8 °C (37.4–38.5 °C)] despite the former having a lower reference range (*U* = 10100, *P* < 0.001) and pyrexia was significantly more common within the donkey population (*χ*^2^ = 58.1, *df* = 1, *P* < 0.001) (Fig. 5). Tachypnoea was also more common in the donkey population (*χ*^2^ = 7.5, *df* = 1, *P* = 0.006) with a higher presenting respiratory rate (*U* = 5239, *P* = 0.001) than in the horse population.

### Disease phenotype differed between sexes

The data are summarised by sex in Table 2. The study population (selected using the clinical criteria) comprised a larger proportion of female (58%, 144/247) than male animals (42%, 103/247), which was more marked than that of the general sampled population (54–46%; *n* = 641). Therefore, both the total and selected populations had increased female: male ratio. Infection status (by PCR) was comparable between sexes.

**Table 2:** Demographics, history and clinical examination findings of population selected on clinical grounds for inclusion in the study (*n* = 247) broken down by sex

	Study population (*N* = 247)	Female (*N* = 144)	Male *N* = 103
Demographic data			
Age (years), median (IQ range)	6 (2.5–12)	6 (2.5–12)	6 (2.5–12)
Donkey, *n*/*N* (%)	144/247 (58)	81/144 (56)	60/103 (58)
Horse, *n*/*N* (%)	103/247 (42)	63/144 (44)	43/103 (42)
Estimated weight (kg)	150 (120–225)	151 (120–227)	149 (115–225)
History			
No veterinary treatment > 1 yr, *n*/*N* (%)	130/241 (54)	71/140 (49)	59/101 (58)
No trypanocidal treatment for > 1 yr, *n*/*N* (%)	149/241 (62)	79/140 (56)	70/101 (69)
Diarrhoea, *n*/*N* (%)	10/247 (4)	3/144 (2)	7/103 (7)
Recent/recurrent colic, *n*/*N* (%)	4/247 (2)	2/144 (1)	2/103 (2)
Body condition score (0–5/5), median (IQ range)	1.5 (1.5–2.0)	1.5 (1.0–1.5)	1.5(1.5–2.0)
0.5, *n*/*N* (%)	18/243 (7)	13/142 (9)	5/101 (5)
1, *n*/*N* (%)	42/243 (17)	29/142 (20)	13/101 (13)
1.5, *n*/*N* (%)	109/243 (45)	68/142 (48)	41/101 (41)
≥ 2, *n*/*N* (%)	74/243 (30)	32/142 (23)	42/101 (42)
Demeanour			
BAR, *n*/*N* (%)	40/224 (18)	18/131 (14)	22/94 (23)
QAR, *n*/*N* (%)	136/224 (61)	86/131 (66)	50/94 (53)
Dull, *n*/*N* (%)	48/224 (21)	27/131 (21)	21/94 (22)
Parameter			
Temperature (°C), median (IQ range)	38.1 (37.7–38.6)	37.7(37.3–38.3)	38.0 (37.6–38.6)
Pulse (bpm), median (IQ range)	56 (48–64)	56 (48–64)	56 (48–64)
Respiration (bpm), median (IQ range)	32 (24–46)	32 (24–44)	32 (28–48)
Haematocrit (%), median (IQ range)	22 (18–24)	22 (19–24)	21 (16–24)
Total plasma protein (g/l), median (IQ range)	78 (70–88)	79 (72–88)	78 (70–88)

*Notes*: Data are presented as median values (interquartile range) or proportions (percentages)

*Abbreviations*: BAR, bright, alert and responsive; QAR, quiet, alert and responsive; IQ, interquartile, n, number of animals with variable present; N, number of animals with variable measured; bpm, breaths or beats per minute

#### Male animals were in better body condition

**Fig. 6:**
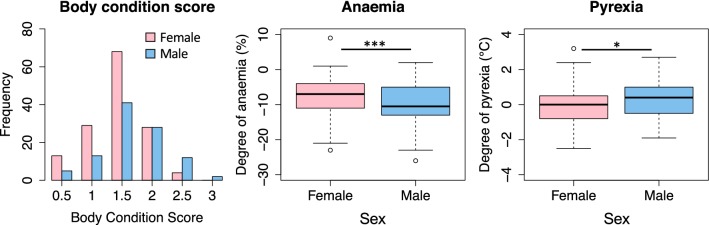
Sex variation in disease phenotype. The differences in data distribution for body condition score (BCS) by sex (*P* < 0.001), in the degree of pyrexia (°C) of the study population (*n* = 247) by sex (*P* < 0.001), and the degree of anaemia (%) of the *Trypanosoma* spp.-positive study population (*n* = 162) by sex (*P* = 0.03). Degree of pyrexia (°C) was calculated as difference between the animal’s rectal temperature and upper reference value for rectal temperature (horse 38.5 °C; donkey 37.8 °C). Degree of anaemia (%) was calculated as difference between the animal’s HCT and the lower reference value (horse 31%; donkey 27%). ***P < 0.001, **P < 0.01, *P < 0.05

Within the male population there was a greater proportion of animals (*χ*^2^ = 10.1, *df* = 1, *P* = 0.001) with an acceptable body condition score (≥ 2) than female animals (Fig. 6, Table 2).

#### Male animals were more febrile

On comparison of clinical parameters, male animals had a higher rectal temperature than females (*U* = 9498, *P* < 0.001) and a greater proportion was classified as pyrexic (*χ*^2^ = 5.96, *df* = 1, *P* = 0.01) (Fig. 6). For animals that were PCR *Trypanosoma* spp.-positive the difference in median rectal temperature between male [38.5 °C (37.9–39.2 °C)] and female animals [37.9 °C (37.5–38.4 °C)] was greater than that observed in the complete study population (*U* = 5538, *P* < 0.001).

#### Male animals were more anaemic

On comparison of clinicopathological parameters of the selected study population male animals had a trend to being more anaemic (*P* = 0.05) (Table 2) and when this analysis was performed using the PCR *Trypanosoma* spp.-positive animals only, the male animals were significantly more anaemic (*U* = 2541, *P* = 0.025) than the female animals (Fig. 6).

## Discussion

This study represents the most detailed evaluation to date of a population of working equids with a naturally acquired trypanosome infection. The data provide both further evidence of the extremely high morbidity of trypanosomiasis in an endemic region and a marked diversity in equid host disease phenotype that warrants further investigation.

On evaluation of the general Gambian equid population, health indices were consistent with a population experiencing high morbidity and mortality. Overall health status was poor, as evidenced by the low median population body condition, frequent aberrant clinical and clinicopathological parameters, and commonly subdued demeanour seen during this study. Furthermore, access to health care in this region was limited. The median population age encountered in the equids was young compared to equine populations surveyed in other regions [45] and together with the positive skew of the equine age distribution, was consistent with a low life expectancy. The findings here are supported by previous studies demonstrating that the mortality rate of equids in The Gambia exceeds foaling rates [16]. It is likely that this situation could be exacerbated by the very high prevalence of reported abortions identified in this study. The unusual sex distribution in a population of working equids in which there is no known sex-specific selection pressure is also interesting. Previous studies evaluating wild equid populations have reported a sex ratio close to 1 [46]. The higher ratio of females to males in this general population could reflect decreased survival of males, and greater morbidity from infectious disease processes, as witnessed in the current investigation, may be one contributory factor. Whilst the challenges to this working equid population are certainly multifactorial, the findings of this study corroborate recommendations for necessary improvements in diagnosis, management and treatment strategies for the hyperendemic trypanosomiasis impacting survival in this location [12, 13, 15].

This study highlights pitfalls in the sensitivity of current gold standard [47] diagnostic tests and suggests that trypanocidal treatment effect may be the strongest indicator of true infection status. Evaluation of the response of the PCR-negative population to trypanocidal treatment would suggest that the threshold of these PCR primers was too high to detect all clinically significant infections and that the true prevalence in this population approached 100%. This may be due to the extremely low parasitaemia present in chronic disease, especially for *T. vivax* and *T. brucei*, which have a significant tissue reservoir [48, 49].

These data also support a marked diversity in the individual (intra-species) disease phenotype of equid trypanosomiasis; clinical expression of disease did not correlate well with *Trypanosoma* spp. PCR status. Previous documentation of PCR prevalence being similar between symptomatic and asymptomatic equids would support this statement [12]. Multiple variables such as disease state (acute or chronic), host susceptibility, pathogenicity and species of parasite and co-infection status could contribute to this finding.

Within this population, parasite co-infections with multiple species of trypanosome (consistent with previous observations [12, 13]) and with *Theileria equi* were common. Uneven distribution of disease occurred, with 0–4 co-infections detected in the study population. This could be due a variety of factors including chance, variation in local disease prevalence and/or host factors. The impact to the host of *Trypanosoma* spp. co-infections is a largely unexplored area. In equines the pathogenicity of *T. vivax* West has been queried, and it has been suggested that infection with *T. vivax* may reduce the pathogenic impact of *T. congolense* [12]. Interactions between specific parasites impacting on disease phenotype were not evident from these clinical data but there was a trend for increasing morbidity with number of co-infections (including *Theileria equi*) suggesting a complex relationship. Available information suggests that interactions between *Trypanosoma* spp. are multifaceted and exist not only at an inter-species [50] but also at an intra-species level [51], having important implications for the pathogenicity of disease to the host.

The contribution of piroplasmosis (*Theileria equi*) to population morbidity in this study was difficult to quantify. Clinical signs of acute and chronic piroplasmosis are well described. In endemic populations a level of immunity is reported in healthy mature animals, but it is probable that the disease still contributes towards a chronic anaemia [52]. In an immunosuppressed population facing multiple co-morbidities, such as The Gambian equid population studied, symptoms are likely to be more prevalent, but clinically indistinguishable from *Trypanosoma* spp. infection. In other species co-infections of *Trypanosoma* spp. and piroplasms have synergistic [53] or antagonistic [54] effects but these infection interactions were not detected in the present study. Quantitative information about the level of parasitaemia, pathogenicity or number of strains present could have enabled further analysis to assess the clinical impact and dynamics of co-infections.

Whilst equine inter-species variability can be influenced by multiple factors, variability within subpopulations implicates host factors. Two important themes were evident within this data analysis: the impact of sex and species of equid on disease phenotype.

The results of this study support a greater resilience to *Trypanosoma* spp. infection in donkeys compared to horses providing objective data to support previous subjective observations [12, 26]. Historical evidence documents the problems encountered during repeated attempts to introduce the horse to the African continent which have been largely attributed to *Trypanosoma* spp. infection [55]. In the present study, horses had more frequently received trypanocidal medication within the last year suggesting more frequent owner concerns of disease. Given horses are more expensive to purchase [21] it would be anticipated that there would be greater investment in their management to optimise health and productivity. Conversely signs of disease were more severe in the horse population; there was a lower presenting body condition score and a greater degree of anaemia than in the donkey population. However, donkeys were more frequently *Trypanosoma* spp.-positive than horses; this contrasts with previous findings [12, 13] but the present study may be more representative due to a larger sample size. Donkeys were also more frequently pyrexic and co-infected with multiple species of trypanosome (consistent with previous observations [12, 13]) than horses. These findings oppose theories of donkey resilience being related to ecological species differences resulting in a reduced feeding frequency of tsetse flies and therefore a lower incidence of trypanosome infection [12]. The higher prevalence of infections observed here, may be due to differing (extensive) management and increased exposure of donkeys to tsetse fly in this region [20].

In livestock, an ability to be more resistant to the effects of a trypanosome infection has been defined by two mechanisms, control of parasitaemia and control of anaemia [56]. The data reported suggest the donkey has better control of anaemia than horses but not necessarily of the level of parasitaemia. In this study, the degree of pyrexia was greater within the donkey population and most marked in PCR-positive animals, supporting an association with *Trypanosoma* spp. infection and a potential correlation with the level of parasitaemia. The reported labile nature of donkey rectal temperature [57, 58] can be countered by the constant environmental temperature and the large number of normothermic donkeys (175/360, 49%) in the whole sampled population. Pyrexia is a common finding in acute *Trypanosoma* spp. infections of donkeys [59] and horses [60]. Total plasma protein, a crude proxy for humoral immune response, was comparable between species. The difference could, therefore, represent a more marked cell-mediated immune response to *Trypanosoma* spp. within the donkey population warranting further investigation.

Disease phenotype also differed between sexes. There was reversal of the normal sex bias when compared to the demographics reported in other equine populations [45] with greater numbers of female animals than male animals. This may support a greater mortality of male animals, consistent with the lower median age of male [4.5 years-old (2–9 years-old)] than female animals [6 years-old (2.5–11 years-old)] in the general sampled equid population. Within the study population male animals were consistently more pyrexic, and this difference was greater within the *Trypanosoma* spp. PCR-positive population. Male *Trypanosoma* spp. PCR-positive animals were also more anaemic than female animals. Infection status was comparable between sexes with except for *T. brucei*, which was more prevalent in males. Male animals were in marginally better body condition than the female animals, but this may be confounded by the significant additional metabolic drains placed upon the female population due to pregnancy and lactation, especially in the context of the high abortion rate. Prevalence and intensity of parasite infections are frequently higher in males than females [61, 62] and this has previously been documented in *Trypanosoma* spp. in male cattle [63] and donkeys [64]. Two aetiologies have been proposed; first, differing behaviours (e.g. risk-taking is more prevalent in males) or appearance can increase exposure to the parasite (ecological factors) and secondly, physiological factors. The major documented physiological factor is the association between testosterone and immuno-suppressive effects [65]. In this study, the total plasma protein concentration was comparable between sexes, but further evaluation of immune function was not performed. Further work is required to establish the importance of the difference at the population level. If the sex effect was significant and physiological, routine castration of non-breeding equines would be a practical application of the observation that could improve population health.

## Conclusions

This study provides evidence of diversity of clinical signs and variable disease phenotypes within subpopulations of infected working equids. PCR status was not associated with a defined disease phenotype and there was both intra- and inter-species variability in disease presentation between donkeys and horses. The results provide clinical data to support the necessity for further exploration of the impact of sex and equid species on the expression of trypanosomiasis disease phenotype and the consequences of parasitic co-infection in this population.

## Supplementary information

**Additional file 1: Figure S1.** Image of the form to structure and record history taking, clinical examination and treatment.

**Additional file 2: Table S1.** Summary of the doses and routes of administration selected for the three trypanocides used (melarsomine dihydrochloride, diminazene and isometamidium). These were based upon the current evidence base for efficacy whilst trying to minimise the probability of complications.

**Additional file 3: Text S1.** Methods for PCR as described previously [31].

**Additional file 4: Table S2.** Summary of the species-specific reference ranges used for description and analysis of clinical and clinicopathological parameters.

**Additional file 5: Table S3.** Summary of the demographics, history and clinical examination findings of whole examined population (*n* = 641). Data are presented as median values (interquartile range) or proportions (percentages).

**Additional file 6: Table S4.** Summary of the demographics, history and clinical examination findings of whole study population (*n* = 247) subdivided by PCR *Trypanosoma* spp. status. Data are presented as median values (interquartile range) or proportions (percentages).

**Additional file 7: Table S5.** Mixed effect GLM model for factors associated with presenting degree of anaemia (%) within the selected study population (*n* = 247). The coefficients indicate the decrease in haematocrit associated with an incremental increase in the continuous variable or alternative status (binary or categorical variable) within this population.

**Additional file 8: Table S6.** Mixed effect GLM model for factors associated with presenting degree of pyrexia (°C) within the selected study population (*n* = 247). The coefficients indicate the increase in rectal temperature associated with an incremental increase in the continuous variable or alternative status (binary or categorical variable) within this population.

## Data Availability

Data supporting the conclusions of this article are provided within the article and its Additional files. The datasets used and/or analysed during the current study are available from the corresponding author upon reasonable request.

## Abbreviations

HCT: haematocrit
TP: total plasma protein
EDTA: ethylenediaminetetraacetic acid
BCS: body condition score
BAR: bright, alert, responsive
QAR: quiet, alert, responsive
IQR: interquartile range
GLM: generalised linear model
