# Supplementary material for: Variation in disease phenotype is marked in equine trypanosomiasis
Source: Parasit Vectors. 2020 Mar 21;13:148. doi: 10.1186/s13071-020-04020-6 (PMC7085162; doi:10.1186/s13071-020-04020-6)
Supplement: Supplementary file 1 — Additional file 1: Figure S1. Image of the form to structure and record history taking, clinical examination and treatment. [file 13071_2020_4020_MOESM1_ESM.pdf]

WEEK 1 EXAMINATION FORM:

Number: [ ] Equine: Donkey/ horse
Owner name: [ ] Owner mob number: [ ]
Age: [ ] yrs Sex: Mare/ Stallion/ Gelding
Colour: [ ] Features: [ ]
Weight: [ ] kg Body condition score: [ ] /5

History

[ ] Health check [ ] Concern ____________

Weight loss?	↑	wnl	↓	Appetite?	↑	wnl	↓
Activity?	↑	wnl	↓	Water intake?	↑	wnl	↓

Abortion? [ ] Previous foals? [ ]
Treated? [ ] ____________

Clinical exam

Attitude: BAR / QAR / Dull / Recumbent
T= [ ] °C P= [ ] bpm R= [ ] bpm
MM [ ] CRT [ ] secs
Oedema?: Ventral/ Limb/ Scrotal/ None
Pregnant?: Yes/ No/ Unknown
Other:

Blood sample [ ]
PCV: [ ] % TP: [ ] g/l
Inclusion? Yes/ No
Microchip: STICKER
Treatment: Cymelarsan/ Diminazene/ Isometamidium [ ] mls
Side effects:

WEEK 2 EXAMINATION FORM:

Number: [ ] Equine: Donkey/ horse
Owner name: [ ] Scan microchip: [ ]
Body condition score: [ ] /5 Check ID [ ]

History

Owners opinion: Improved No change Deteriorated

Weight loss?	↑	wnl	↓	Appetite?	↑	wnl	↓
Activity?	↑	wnl	↓	Water intake?	↑	wnl	↓

Abortion? [ ]
Side effects?

Clinical exam

Attitude: BAR / QAR / Dull / Recumbent
T= [ ] °C P= [ ] bpm R= [ ] bpm
MM [ ] CRT [ ] secs
Oedema?: Ventral/ Limb/ Scrotal/ None
Injection site:
Other:

Blood sample [ ]
PCV: [ ] % TP: [ ] g/l

WEEK 3 EXAMINATION FORM:

Number: [ ] Equine: Donkey/ horse
Owner name: [ ] Scan microchip: [ ]
Body condition score: [ ] /5 Check ID [ ]

History

Owners opinion: Improved No change Deteriorated

Weight loss?	↑	wnl	↓	Appetite?	↑	wnl	↓
Activity?	↑	wnl	↓	Water intake?	↑	wnl	↓

Abortion? [ ]
Side effects?

Clinical exam

Attitude: BAR / QAR / Dull / Recumbent
T= [ ] °C P= [ ] bpm R= [ ] bpm
MM [ ] CRT [ ] secs
Oedema?: Ventral/ Limb/ Scrotal/ None
Injection site:
Other:

Blood sample [ ]
PCV: [ ] % TP: [ ] g/l
