# Supplementary material for: Variation in disease phenotype is marked in equine trypanosomiasis
Source: Parasit Vectors. 2020 Mar 21;13:148. doi: 10.1186/s13071-020-04020-6 (PMC7085162; doi:10.1186/s13071-020-04020-6)
Supplement: Supplementary file 2 — Additional file 2: Table S1. Summary of the doses and routes of administration selected for the three trypanocides used (melarsomine dihydrochloride, diminazene and isometamidium). These were based upon the current evidence base for efficacy whilst trying to minimise the probability of complications. [file 13071_2020_4020_MOESM2_ESM.docx]

## Additional file 2: Table S1. Summary of the doses and routes of administration selected for the three trypanocides used (melarsomine dihydrochloride, diminazene and isometamidium).

These were based upon the current evidence base for efficacy whilst trying to minimise the probability of complications.

| **Drug trade name** | **Compound** | **Dose (mg/kg)** | **Route of administration** | **Concentration (%)** |
| --- | --- | --- | --- | --- |
| Diminasan | Diminazene aceturate | 3.5mg/kg | i.m. split into two aliquots one in each rump; using a 20g 1.5 inch needle. | 5% |
| Cymelarsan | Melarsamine dihydrochloride | 0.25mg/kg | i.v. 19g 1.5inch needle. | 0.5% |
| Samorin | Isometamidium chloride | 0.5mg/kg | i.v. 19g 1.5inch needle | 0.5% |
