# Supplementary material for: Variation in disease phenotype is marked in equine trypanosomiasis
Source: Parasit Vectors. 2020 Mar 21;13:148. doi: 10.1186/s13071-020-04020-6 (PMC7085162; doi:10.1186/s13071-020-04020-6)
Supplement: Supplementary file 3 — Additional file 3: Text S1. Methods for PCR as described previously [31]. [file 13071_2020_4020_MOESM3_ESM.docx]

## Additional file 3: Text S1. Methods for PCR as described previously [1]

### DNA extraction

Genomic DNA was extracted from the whole blood samples using QIAamp DNA Blood Maxi Kit (QIAGEN®) standardised technique [2] (with the substitution of 50 µl Proteinase K (20 mg/ml) for the supplied Qiagen Protease®) eluting a volume of 600 µl. In the absence of whole blood samples, genomic DNA was extracted from the Whatman® FTA® cards using the QIAamp DNA Micro Kit (QIAGEN®) for gDNA extraction [3]. To compensate for the uneven distribution of trypanosomes across a blood spot on an FTA card [4] a total of 9 x 2mm punches were taken from each card to determine infective status for each sample. For a given individual the same type of sample was used for each time point.

### PCR analysis

To confirm the presence of equine DNA (*Equus ferus caballus* or *Equus africanus asinus*) in the samples following DNA extraction a species specific primer targeting a highly conserved equine cytochrome b sequence was used [5].

To detect *Trypanosoma* sp*.* DNA, primers [6] targeting the multicopy number satellite DNA located on the mini chromosomes, surrounding the genes coding for the variable surface glycoprotein, were selected due to the high sensitivity and specificity. The *Trypanosoma sp.* and subspecies were selected based upon previous surveys in this region [7,8]. These primers, *Trypanosoma congolense* Savannah (TCS 1 and 2), *Trypanosoma brucei* sp*.* (TBr1 and 2) and *Trypanosoma vivax* West (TVW1 and 2) are the current gold standard diagnostic as recommended by the OIE [9].

PCR amplification was conducted in a total reaction volume of 25 µl comprising 2.5 µl template DNA, 1 x HP buffer, 0.25 mM dNTPs, 1.5mM MgCl_2_, 0.01 µg/µl Fw primer, 0.01 µg/µl Rv primer and 1.25U Taq Thermo Hotstart. Positive and negative controls were used to minimise the probability of unidentified false positive or negative results. Positive controls were stock samples of laboratory strains (*T. brucei* 13/13 404; *T. congolense* gifted L.M., *T. vivax* gifted L.M.). Reaction conditions were maintenance at 95⁰C for 15 min followed by 32 cycles of amplification (Techne® PCR machine). This was followed by denaturation at 94⁰C for 15 s, annealing at 60⁰C for 30 s, extension at 72⁰C for 90 s. The PCR solution was then held at 72⁰C for 10 min. The melting temperature for TBr1 and TBr2 is lower necessitating a reduction in annealing temperature of 55⁰C from 60⁰C. For TVW1 and TVW 2 the annealing temperature was increased to 62⁰C and the cycle number was decreased to 30 (for DNA extracted from whole blood) to prevent non-specific binding of primers. Cycle number was increased to 35 cycles (for all *Trypanosoma sp.*) for DNA extracted from FTA cards to account for a lower starting concentration of DNA*.* Following amplification, for each sample 10 µl of PCR product was loaded on to a 2% agarose gel (SeaKem) containing 1 µg/ml ethidium bromide. 5 µl of 100 bp ladder (Invitrogen) was used to assess the PCR product size. The electrophoresis was performed for 45 min at 150V in 1 x TBE buffer prior to examination and imaging in a UV transilluminator.

**References**

1. Raftery AG, Jallow S, Rodgers J, Sutton DGM. Safety and efficacy of three trypanocides in confirmed field cases of trypanosomiasis in working equines in The Gambia: a prospective, randomised, non-inferiority trial. PLoS Negl Trop Dis. 2019;13:e0007175.

2. QIAamp DNA Blood Midi/Maxi Handbook - QIAGEN [Internet]. [cited 2017 Oct 18]. Available from: https://www.qiagen.com/gb/resources/resourcedetail?id=bf32146a-77fd-40c2-8743-c28974f7935b&lang=en

3. QIAamp DNA Micro Handbook - QIAGEN [Internet]. [cited 2017 Oct 18]. Available from: https://www.qiagen.com/gb/resources/resourcedetail?id=085e6418-1ec0-45f2-89eb-62705f86f963&lang=en

4. Cox AP, Tosas O, Tilley A, Picozzi K, Coleman P, Hide G, et al. Constraints to estimating the prevalence of trypanosome infections in East African zebu cattle. Parasit Vectors. 2010;3:82.

5. Matsunaga T, Chikuni K, Tanabe R, Muroya S, Shibata K, Yamada J, et al. A quick and simple method for the identification of meat species and meat products by PCR assay. Meat Sci. 1999;51:143–8.

6. Masiga DK, Smyth AJ, Hayes P, Bromidge TJ, Gibson WC. Sensitive detection of trypanosomes in tsetse flies by DNA amplification. Int J Parasitol. 1992;22:909–18.

7. Duffy CW, Morrison LJ, Black A, Pinchbeck GL, Christley RM, Schoenefeld A, et al. Trypanosoma vivax displays a clonal population structure. Int J Parasitol. 2009;39:1475–83.

8. Pinchbeck GL, Morrison LJ, Tait A, Langford J, Meehan L, Jallow S, et al. Trypanosomosis in The Gambia: prevalence in working horses and donkeys detected by whole genome amplification and PCR, and evidence for interactions between trypanosome species. BMC Vet Res. 2008;4:7.

9. OIE. Manual of Diagnostic Tests and Vaccines for Terrestrial Animals 2017 [Internet]. [cited 2016 Dec 27]. Available from: http://www.oie.int/en/international-standard-setting/terrestrial-manual/access-online/
