# Supplementary material for: Variation in disease phenotype is marked in equine trypanosomiasis
Source: Parasit Vectors. 2020 Mar 21;13:148. doi: 10.1186/s13071-020-04020-6 (PMC7085162; doi:10.1186/s13071-020-04020-6)
Supplement: Supplementary file 4 — Additional file 4: Table S2. Summary of the species-specific reference ranges used for description and analysis of clinical and clinicopathological parameters. [file 13071_2020_4020_MOESM4_ESM.docx]

## Additional file 4: Table S2. Species specific reference ranges used for description and analysis of clinical and clinicopathological parameters [1–4]

| **Parameter** | **Donkey** | **Horse** |
| --- | --- | --- |
| Rectal temperature (˚C) | 36.2-37.8 | 37.5-38.5 |
| Heart rate (bpm) | 40-53 | 24-40 |
| Respiratory rate (bpm) | 16-20 | 8-16 |
| Haematocrit (%) | 27-42 | 31-43 |

**References**

1. Duncan J, Hadrill D. The professional handbook of the donkey. Duncan J, Hadrill D, editors. Yatesbury, UK: Whittet Books Limited; 2008.

2. Reed, SM, Bayly, WM, Sellon, DC. Equine Internal Medicine. 3 edition. St. Louis, Mo.: Saunders; 2009.

3. Burden FA, Hazell-Smith E, Mulugeta G, Patrick V, Trawford R, Brooks Brownlie HW. Reference intervals for biochemical and haematological parameters in mature domestic donkeys (Equus asinus) in the UK. Equine Vet Educ. 2016;28:134–9.

4. Clinicopathological Reference Ranges Adult Horses, Rossdales Laboratory [Internet]. Rossdales. [cited 2017 Apr 25]. Available from: https://www.rossdales.com/laboratories/reference-ranges/
