# Supplementary material for: Variation in disease phenotype is marked in equine trypanosomiasis
Source: Parasit Vectors. 2020 Mar 21;13:148. doi: 10.1186/s13071-020-04020-6 (PMC7085162; doi:10.1186/s13071-020-04020-6)
Supplement: Supplementary file 5 — Additional file 5: Table S3. Summary of the demographics, history and clinical examination findings of whole examined population (n = 641). Data are presented as median values (interquartile range) or proportions (percentages). [file 13071_2020_4020_MOESM5_ESM.docx]

**Additional file 5: Table S3.** Demographics, history and clinical examination findings of whole examined population (n=641)

|  | **Whole population**  **(*N* = 641)** | **Donkeys**  **(*N* = 362)** | **Horses**  **(*N* = 279)** |
| --- | --- | --- | --- |
| Demographic data |  |  |  |
| Age (years), median (IQ range) | 5 (2-10) | 5 (2.5-8) | 6 (2-12) |
| Female, *n/N* (%) | 342/637 (54) | 198/359 (55) | 144/276 (52) |
| Male, *n/N* (%) | 295/637 (46) | 163/359 (45) | 132/276 (48) |
| Estimated weight (kg), median (IQ range) | 145(115-225) | 123(110-145) | 229 (180-260) |
| History |  |  |  |
| No trypanocidal treatment for >1yr, *n/N* (%) | 418/641 (65) | 270/362 (75) | 148/279 (53) |
| Abortion, *n/N* (%) | 60/340 (18) | 28/196 (14) | 32/144 (22) |
| Diarrhoea, *n/N* (%) | 17/639 (3) | 3/362 (0.8) | 14/279 (5) |
| Recent/ recurrent colic, *n/N* (%) | 19/639 (3) | 2/362 (0.6) | 17/279 (6) |
| Body condition score (0-5/5), median (IQ range) | 2(1.5-2) | 2 (1.5-2) | 1.5(1.5-2) |
| 0.5, *n/N* (%) | 24/622 (4) | 3/352 (0.9) | 21/268 (8) |
| 1, *n/N* (%) | 57/622 (9) | 23/352 (7) | 34/268 (13) |
| 1.5, *n/N* (%) | 203/622 (33) | 95/352 (27) | 108/268 (40) |
| ≥2, *n/N* (%) | 338/622 (54) | 231/352 (66) | 105/268 (39) |
| Demeanour |  |  |  |
| BAR, *n/N* (%) | 216/565 (38) | 148/317 (47) | 69/247 (28) |
| QAR, *n/N* (%) | 286/565 (51) | 140/317 (44) | 144/247 (58) |
| Dull, *n/N* (%) | 63/565 (11) | 29/317(9) | 34/247 (14) |
| Parameters |  |  |  |
| Temperature (°C), median (IQ range) | 37.8(37.5-38.3) | 37.9 (37.6-38.3) | 37.8(37.4-38.2) |
| Pyrexia, *n/N* (%) | 215/628 (34) | 177/354 (50) | 38/274 (14) |
| Pulse (bpm) | 54(44-60) | 56(52-64) | 48(40-56) |
| Tachycardia, *n/N* (%) | 425/633 (67) | 221/358 (62) | 204/275 (74) |
| Respiration (bpm), median (IQ range) | 32(24-40) | 32(28-44) | 28(24-36) |
| Haematocrit (%), median (IQ range) | 26(23-30) | 26(23-29) | 26(23-30) |
| Anaemia, *n/N* (%) | 404/612 (66) | 183/337 (54) | 221/275 (80) |

*Notes*: Data are presented as median values (interquartile range) or proportions (percentages)

*Abbreviations*: BAR, bright, alert and responsive; QAR, quiet, alert and responsive; IQ, interquartile, n, number of animals with variable present; N, number of animals variable measured; bpm, breaths or beats per minute.
