# Supplementary material for: Variation in disease phenotype is marked in equine trypanosomiasis
Source: Parasit Vectors. 2020 Mar 21;13:148. doi: 10.1186/s13071-020-04020-6 (PMC7085162; doi:10.1186/s13071-020-04020-6)
Supplement: Supplementary file 6 — Additional file 6: Table S4. Summary of the demographics, history and clinical examination findings of whole study population (n = 247) subdivided by PCR Trypanosoma spp. status. Data are presented as median values (interquartile range) or proportions (percentages). [file 13071_2020_4020_MOESM6_ESM.docx]

## Additional file 6: Table S4. Demographics, history and clinical examination findings of whole study population (n=247) subdivided by PCR *Trypanosoma* spp*.* status.

| **Variable** | ***Trypanosoma* spp*.* PCR-positive n=162** | ***Trypanosoma* spp*.* PCR-negative n=85** |
| --- | --- | --- |
| Presenting for health concern, *n/N* (%) | 111/147 (76) | 47/79 (59) |
| Age (years), median (IQ range) | 7 (2.5-11.0) | 5 (2.5-13.0) |
| Sex |  |  |
| Female, *n/N* (%) | 94/162 (58) | 50/85 (59) |
| Male, *n/N* (%) | 68/162 (42) | 35/85 (41) |
| Species |  |  |
| Horse, *n/N* (%) | 55/162 (34) | 51/85 (60) |
| Donkey, *n/N* (%) | 107/162 (66) | 34/85 (40) |
| Clinical examination |  |  |
| BCS (0-5/5), median (IQ range) | 1.5 (1-2) | 1.5 (1.5-2) |
| Temperature (°C), median (IQ range) | 38.2 (37.7-38.8) | 38.0 (37.5-38.5) |
| Heart rate (bpm), median (IQ range) | 59 (50-64) | 52 (44-62) |
| Respiratory rate (bpm), median (IQ range) | 32 (24-44) | 36 (24-48) |
| Haematocrit (%), median (range) | 20 (5-36) | 23 (12-38) |

*Notes*: Data are presented as median values (interquartile range) or proportions (percentages)

*Abbreviations*: BAR, bright, alert and responsive; QAR, quiet, alert and responsive; IQ, interquartile, n, number of animals with variable present; N, number of animals variable measured; bpm, breaths or beats per minute.
