# Supplementary material for: Variation in disease phenotype is marked in equine trypanosomiasis
Source: Parasit Vectors. 2020 Mar 21;13:148. doi: 10.1186/s13071-020-04020-6 (PMC7085162; doi:10.1186/s13071-020-04020-6)
Supplement: Supplementary file 7 — Additional file 7: Table S5. Mixed effect GLM model for factors associated with presenting degree of anaemia (%) within the selected study population (n = 247). The coefficients indicate the decrease in haematocrit associated with an incremental increase in the continuous variable or alternative status (binary or categorical variable) within this population. [file 13071_2020_4020_MOESM7_ESM.docx]

## Additional file 7: Table S5. Mixed effect GLM model for factors associated with presenting degree of anaemia (%) within the selected study population (n=247). The coefficients indicate the decrease in haematocrit associated with an incremental increase in the continuous variable or alternative status (binary or categorical variable) within this population.

|  | **Estimate** | **Std. Error** | **Chisq** | **P value** |
| --- | --- | --- | --- | --- |
| (Intercept) | -2.90 | 1.13 |  |  |
| Tachycardia (bpm) | -0.07 | 0.03 | 7.1 | <0.001 |
| Species (Horse) | -3.06 | 0.71 | 16.9 | <0.001 |
| *T. brucei* status week 1 | -2.35 | 0.87 | 8.7 | 0.003 |
| *T. congolense* status week 1 | -1.90 | 0.65 | 8.8 | 0.003 |
| *T. vivax* status week 1 | -1.69 | 0.65 | 6.2 | 0.010 |
| Attitude Dull | -2.86 | 0.95 | 10.7 | 0.005 |
| Attitude_QAR | -0.60 | 0.80 |  |  |

*Abbreviations*: QAR, quiet, alert and responsive; bpm, breaths or beats per minute.
