# Supplementary material for: Variation in disease phenotype is marked in equine trypanosomiasis
Source: Parasit Vectors. 2020 Mar 21;13:148. doi: 10.1186/s13071-020-04020-6 (PMC7085162; doi:10.1186/s13071-020-04020-6)
Supplement: Supplementary file 8 — Additional file 8: Table S6. Mixed effect GLM model for factors associated with presenting degree of pyrexia (°C) within the selected study population (n = 247). The coefficients indicate the increase in rectal temperature associated with an incremental increase in the continuous variable or alternative status (binary or categorical variable) within this population. [file 13071_2020_4020_MOESM8_ESM.docx]

**Additional file 8: Table S6.** Mixed effect GLM model for factors associated with presenting degree of pyrexia (°C) within the selected study population (n=247). The coefficients indicate the increase in rectal temperature associated with an incremental increase in the continuous variable or alternative status (binary or categorical variable) within this population.

|  | **Coefficients** | **Std. Error** | **Chisq** | **P value** |
| --- | --- | --- | --- | --- |
| (Intercept) | -0.35 | 0.168 |  |  |
| Tachycardia (bpm) | 0.03 | 0.004 | 28.8 | <0.001 |
| Sex (Male) | 0.40 | 0.095 | 22.5 | <0.001 |
| Species (Horse) | -0.93 | 0.107 | 74.6 | <0.001 |
| *T. brucei* status week 1 | 0.30 | 0.133 | 4.0 | 0.046 |
| Respiration (bpm) | 0.02 | 0.004 | 15.5 | <0.001 |
| Estimated age (years) | -0.02 | 0.008 | 8.7 | 0.003 |

*Abbreviations*: bpm, breaths or beats per minute.
